# Non-coding RNA: a potential biomarker and therapeutic target for sepsis

**DOI:** 10.18632/oncotarget.21766

**Published:** 2017-10-10

**Authors:** Tie-Ning Zhang, Da Li, Jing Xia, Qi-Jun Wu, Ri Wen, Ni Yang, Chun-Feng Liu

**Affiliations:** ^1^ Department of Pediatrics, PICU, Shengjing Hospital of China Medical University, Shenyang, China; ^2^ Department of Obstetrics and Gynecology, Shengjing Hospital of China Medical University, Shenyang, China; ^3^ Department of Clinical Epidemiology, Shengjing Hospital of China Medical University, Shenyang, China

**Keywords:** sepsis, non-coding RNA, biogenesis, biomarkers, therapeutic target

## Abstract

Sepsis, a syndrome of physiologic, pathologic, and biochemical abnormalities caused by an altered systemic host response to infection, has become the main cause of death among patients admitted to the intensive care units. Recently, genome-wide expression analysis revealed that over 80% of the essential genetic elements were altered in critically ill patients. Notably, non-coding RNAs, including microRNAs, long non-coding RNAs and circular RNAs, have been proven to play essential roles in innate immunity, mitochondrial dysfunction and organ dysfunction. In this review, we introduced the biogenesis of non-coding RNAs briefly and summed up different kinds of non-coding RNAs in regulation of sepsis, which could provide a more comprehensive understanding about pathogenesis of the disease. Additionally, we summarized the limitations of current biomarkers and then recommended some non-coding RNAs as novel potential biomarkers for sepsis and sepsis-induced organ dysfunction. Besides, we also introduced some problems and challenges that need to be overcome during the clinical application of non-coding RNAs. Future research should focus on elucidating their molecular mechanisms, particularly long non-coding RNAs as well as circular RNAs and sepsis, to further understanding of the disease process. With the in-depth understanding of the mechanism of sepsis, non-coding RNAs provide a new insight into sepsis and could become the novel therapeutic targets in the future.

## INTRODUCTION

Sepsis is a syndrome of physiological, pathological and biochemical abnormalities because of an altered systemic host response to infection [1, 2]. Nowadays, sepsis and septic shock have become major public health problems, being the main cause of death among patients admitted to the intensive care units [3, 4]. Each year, millions of people are affected by sepsis and the reported incidence of sepsis is increasing worldwide [3, 5, 6] despite improvements in intensive care treatments. The study conducted by Zhou et al showed that the standardized occurrence rate was 461, 68, and 52 cases per 100,000 populations for sepsis, severe sepsis, and septic shock, respectively [7]. Besides, the study preformed by Martin et al found that there were 10,319,418 reported cases of sepsis (accounting for 1.3 percent of all hospitalizations) during a 22-year study period in the United State [8]. The outcome and prognosis for septic patients are unsatisfactory and clinical studies have showed that in-hospital mortality from septic shock might reach approximately 40% [1, 3]. In addition, patients who survive sepsis often suffer from long-term physical, psychological and cognitive disabilities [9]. Study showed that incident severe sepsis was associated with a clinically and statistically significant increase in moderate to severe cognitive impairment among survivors [9]. Consequently, critical care specialists are aware of this disease, attempting to diagnose it at an early stage and manage septic patients more rationally.

According to the latest guidelines, fluid resuscitation, antimicrobial therapy, vasoactive mediation, and if necessary, a supportive therapy for organ dysfunction are recommended for the treatment of sepsis and septic shock [3]. Moreover, considering sepsis as medical emergencies, the latest guidelines recommend early diagnosis and that treatment should begin immediately [3]. However, the sensitivity and specificity of current biomarkers for the early diagnosis of sepsis, such as C-reaction protein (CRP), procalcitonin (PCT) and interleukin-6 (IL-6) are limited [10, 11], as they have been implicated in other non-inflammatory processes [12]. Recently, genome-wide expression analysis revealed that over 80% of the essential genetic elements were altered in critically ill patients [13]. Notably, a class of non-coding RNAs, including microRNAs (miRNAs), long non-coding RNAs (lncRNAs) and circular RNAs (circRNAs) have been identified as regulators of different signalling pathways and therefore, referred to as regulator RNAs [14]. Based on *in vivo* and *in vitro* experiments, these molecules have been proven to play essential roles in innate immunity, mitochondrial dysfunction and organ dysfunction [15-20]. Consequently, it has been suggested that these non-coding RNAs are potential biomarkers or therapeutic targets for sepsis [21, 22].

Here in, this review will discuss how non-coding RNAs affect the pathogenesis of sepsis and their potential use as biomarkers or therapeutic targets for sepsis.

## BIOGENESIS OF NON-CODING RNA

### Biogenesis of miRNAs

**Figure 1:**
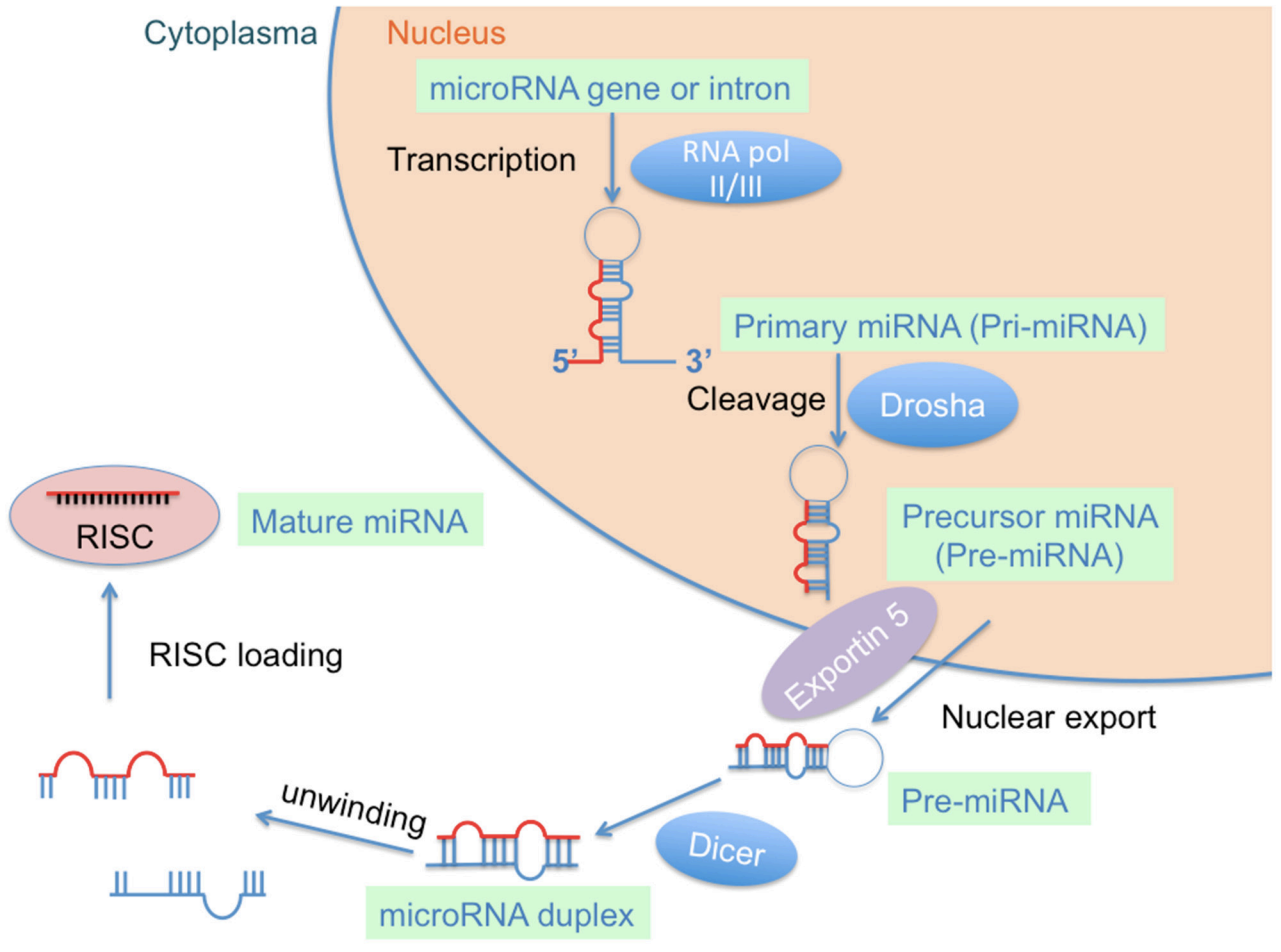
Biogenesis of microRNA (miRNA)

MiRNAs are endogenous non-coding RNA molecules of approximate 19–22 nucleotides in length [23]. The biogenesis of miRNAs has been described in previous reviews [14, 24]. Briefly, they are mainly transcribed by RNA polymerase II resulting in a primary miRNA (called pri-miRNA) with 500–3000 nucleotides [25] (Figure 1). The pri-miRNA is then cleaved into a premature miRNA (called pre-miRNA) 70–80 nucleotides in length by the “microprocessor complex”, which consists of the RNase III Dorsha enzyme and the DiGeorge Syndrome Critical Region 8 (DGCR) protein [26]. The pre-miRNA is exported into the cytoplasm with the help of the nuclear export transporter, exportin 5, processing about 22 nucleotides “miRNA duplex” by interacting with RNase III endonuclease Dicer protein and the co-factor double-stranded transactivation-responsive RNA-binding protein [27]. The miRNA duplex is integrated into the “RNA-induced silencing complex” (RISC) after binding to the argonaute protein and a glycine tryptophan repeat-containing protein, where they bind to partial or full-complementary sequences in the 3’ or 5’ UTR of the target mRNA [28, 29]. MiRNAs have been demonstrated to participate in the comprehensive network of gene regulation in the pathophysiological processes of many diseases [30, 31].

### Biogenesis of lncRNA

LncRNAs are a class of non-protein-coding transcripts larger than 200 nt in length [32]. According to the latest version of LNCipedia, a database for annotated human lncRNA transcript sequences and structures, there are over 60,000 members of the lncRNA family that have been catalogued [33, 34]. Notably, the primary sequence of lncRNAs is poorly conserved, but it can be partially compensated through a high degree of structural conservation [35]. Furthermore, lncRNAs can be transcribed from conserved genomic regions [36] and back-splicing of exons, which could form circRNAs, can also generate lncRNAs [37, 38]. LncRNAs take part in some important functions of cells including chromatin rearrangement, histone modification, and modification of alternative splicing genes, as well as the regulation of gene expression. Therefore, lncRNAs play important role in the pathogenesis of various diseases [39, 40].

### Biogenesis of circRNA

**Figure 2:**
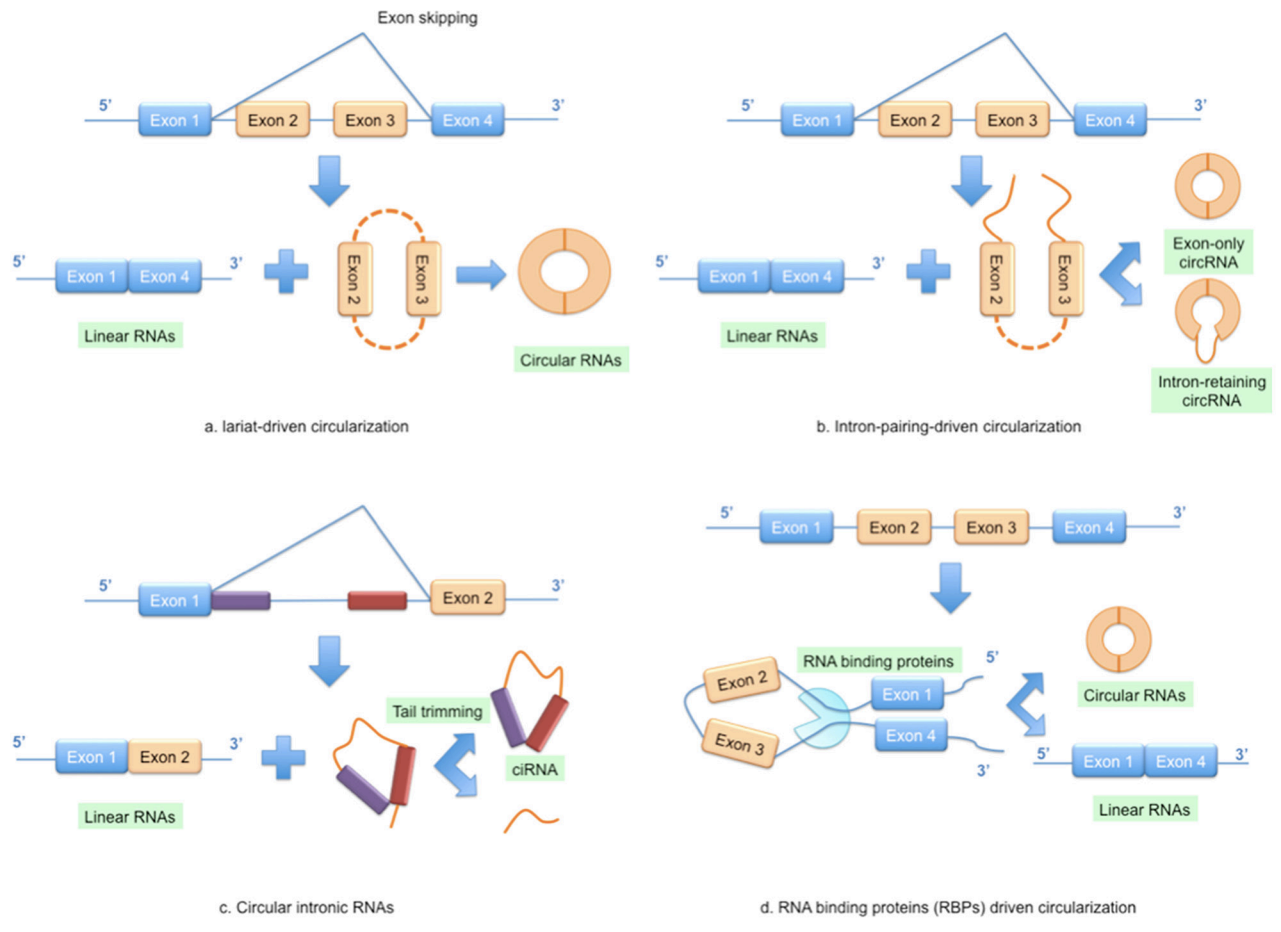
Models of circular RNA (circRNA) biogenesis **(A)** Lariat-driven circularization; **(B)** Intron-pairing-driven circularization; **(C)** Circular intronic RNAs; **(D)** RNA binding proteins (RBPs) driven circularization.

Initially, circRNAs were generally considered to be of low abundance but recent high-throughput sequencing and novel computational approaches have since shown them to be widespread and substantial within transcriptomes [37, 38, 41]. CircRNAs mainly come from the exons of protein-coding genes and they are not formed by the normal model of RNA splicing [42]. They are characterized by covalently closed loop structures through joining the 3’ and 5’ end together by exon circularization or intron circularization [41, 43]. A study conducted by Jeck *et al.* showed that circRNAs were formed via two different mechanisms of exon circularization, lariat-driven circularization and intron-pairing-driven circularization [37] (Figure 2A & 2B). As for introns between exons, when they form a circular structure, they will be removed or retained to form an exon-only circRNA or intron-retaining circRNA called EIciRNA [37, 44] (Figure 2C). Furthermore, circRNAs can also be generated from the circularization of two flanking intronic sequences [45, 46] (Figure 2D). Recently, many circRNAs have been successfully identified in various tissues or organs and some have been linked to disease, suggesting that circRNAs are not simply by-products of mis-splicing or splicing errors [16, 43].

## NON-CODING RNAS IN THE DEVELOPMENT OF SEPSIS

Non-coding RNAs are linked and also appear variational trend during the pathogenesis of sepsis. Up to now, most studies concentrate on the relationship between miRNAs and sepsis, only a few studies have investigated the relationship between lncRNA/circRNA and sepsis [14].

### Regulation of miRNAs in sepsis

It is well-accepted that miRNAs play important roles in the innate and adaptive immunity during the pathogenesis of different diseases [47]. MiRNAs can modulate T helper cell and regulatory T cell development, which are critical in the process of controlling the host response to disease [48, 49]. During the process of sepsis, the host immune system will appear in pro-inflammatory and immunosuppressed states [50, 51]. Several cytokines are produced in pro-inflammatory state during sepsis, such as the representative and most familiar, tumor necrosis factor-α (TNF-α). The production of TNF-α is controlled by miRNAs at transcriptional and translational levels. For example, a study conducted by Dan *et al.* showed that upregulation of miR-181 could enhance TNF-α mRNA degradation [52]. Similarly, Huang *et al.* reported that miR-125b decreased significantly in association with higher TNF-α expression by neonatal monocytes after lipopolysaccharide (LPS) stimulation [53]. Moreover, miRNAs can directly target the TNF pathway and mediate the inflammatory reaction. For instance, Puimege *et al.* demonstrated that miR-511 is a genuine TNF receptor 1 protein and influences TNF sensitivity, partially protecting against TNF dependent endotoxic shock syndrome [54]. Apart from TNF-α, it also has been documented that other pro-inflammatory cytokines such as IL-6 could increase significantly in sepsis patients [55]. Indeed, Zhou *et al.* reported that the down-regulation of miR-146a was associated with increased levels of IL-6 in sepsis patients [56].

**Figure 3:**
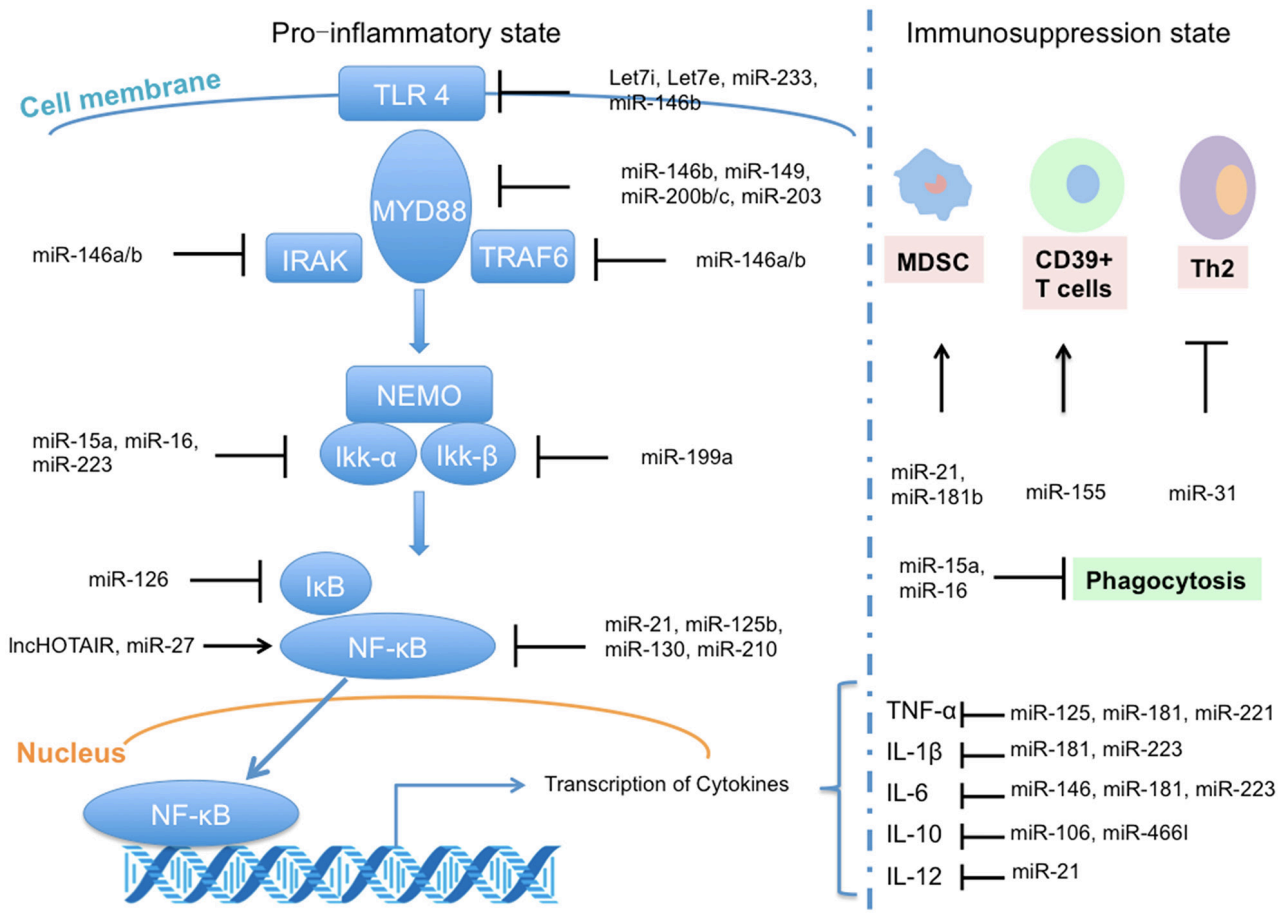
Regulation of microRNA (miRNA) and long non-coding RNA (lncRNA) in sepsis, including interacting with toll-like receptor 4 (TLR4) signal pathway in pro-inflammatory state and regulation functions in immunosuppression state (based on [14])

A number of studies found that miRNAs could regulate inflammation through targeting toll-like receptor (TLR) signalling pathway (Figure 3). TLR mediated signalling mainly activates nuclear factor kappa B (NF-κB), an important transcription factor regulating the expression of immunoregulatory and pro-inflammatory mediators [57-59]. Previous studies showed miRNAs, including miR-146a, miR-125, and miR-155, played critical roles in the negative regulation of TLR/NF-κB mediated innate immune and inflammatory responses [60-62]. Interestingly, transcription of primary miR-155 and several other miRNAs depend on NF-κB [63]. For example, Taganov *et al.* reported that using LPS to stimulate human monocytic THP-1 cells could rapidly induce the expression of both miR-146a and miR-146b [64]. Notably, miR-146a can function on IRAK-1 and TRAF6 directly, which are the key adapter molecules in the TLR signalling pathway [58, 59, 65]. Additionally, miR-146a plays a vital role *in vitro* monocytic cell-based endotoxin tolerance [66, 67] and endotoxin tolerance could be revised by miR-146a inhibition [66]. Recently, Guan *et al.* reported that the NF-κB/DICER signalling pathway suppressed the expression of TNF-α by generating mature forms of miR-125b and miR-130a that negatively regulate TNF-α mRNA [68]. Therefore, TLR-mediated signalling pathway is essential in sepsis [69].

With the progression of sepsis, the immune system will be reprogrammed into the immunosuppression state [50, 70] partly mediated by miRNAs, which can induce immune cell polarization and alter cellular immunity [71-73] (Figure 3). The upregulated expression of miR21 and miR-181b in myeloid-derived suppressor cells in septic mice precludes the differentiation of macrophages and dendritic cells through influencing the transcription factors Stat3 and C/EBPβ [72, 74]. Additionally, there is a temporal regulation in myeloid cells, with miR-155 inversely correlating with BMAL1 levels stimulated by LPS [75]. Also, other studies also proved that various miRNAs, such as mi-R210, miR-23b and miR-29a, could inhibit the expression of NF-κB and IL-6 during sepsis via influencing different immune cells [25, 76, 77]. These studies highlight the profound integration of miRNAs in the pathophysiological of sepsis.

### Regulation of lncRNAs and circRNAs in sepsis

Although a previous review demonstrated that misregulation of lncRNAs could lead to various diseases because lncRNAs were discovered in important biological and development processes [78], there have been only limited studies to evaluate the relationship between lncRNAs and sepsis. So far, several studies have indicated that the expression of lncRNAs changes in human tubular epithelial cells, cardiomyocytes and monocytes during the process of sepsis or exposure to LPS [79-81]. Furthermore, a recent study in human umbilical vein endothelial cells revealed that a number of lncRNAs were upregulated (with a maximum upregulation of about 70 fold), while many lncRNAs were downregulated (with a maximum downregulation of approximately 28 fold) [82]. Furthermore, Cui *et al.* showed that the lncRNA lnc-IL7R, which overlaps with the 3’UTR of the human interleukin-7 receptor α-subunit gene, was significantly upregulated in LPS-treated cells and could regulate inflammatory regulation [83]. However, the role of lncRNAs in sepsis has not been fully elucidated and future research is required to investigate how lncRNAs influence sepsis and determine the exact molecular signalling pathways.

Recently, many circRNAs have been found to act as miRNAs sponges to regulate gene expression [38, 43, 84]. During the process of sepsis, miRNAs are differentially expressed inferring that some circRNAs may be involved. A recent study showed that an LPS-inducible circRNA, mcircRasGEF1B, could regulate the stability of mature ICAM-1 mRNAs and might protect cells against microbial infection [85]. The experimental identification and characterization of circRNAs and their associated molecules, such as miRNAs, is required to gain an insight into the pathogenesis of sepsis and to identify novel biomarkers.

## NON-CODING RNAS AS POTENTIAL BIOMARKERS IN SEPSIS

### Limitations of present biomarkers

According to the National Institute of Health, a biomarker is a “characteristic that can be objectively measured and evaluated as an indicator of normal biological processes, pathological processes, or pharmacological responses to a therapeutic intervention” [86]. Although microbiological culture is the gold standard in distinguishing sepsis from other diseases [3, 87], this method is time-consuming and often associated with false negative results. Additionally, CRP is another common parameter but it can also increase after burns and cannot differentiate between infected and non-infected burn patients [88]. CRP lacks specificity, and concentrations may be increased in other inflammatory and all infectious disorders [12]. Low levels of CRP should be interpreted with caution in patients with fulminant hepatic failure, because these may reflect severity of hepatic synthetic function rather than sepsis or inflammation [89]. Furthermore, PCT is a pro-hormone of calcitonin and may be released from tissues and cells during infection [90]. The guidelines suggest that measurement of PCT levels can be used to support shortening the duration of antimicrobial therapy in septic patients [3]. Although a meta-analysis showed that PCT might be useful to guide antibiotic therapy in septic patients [91], recent studies demonstrated that PCT-guidance therapy did not affect the frequency of diagnostic or therapeutic procedures [3, 92]. Besides, there is no single cut-off range of PCT levels for defining sepsis. The optimum cut-off ranges for PCT depend on many factors such as the clinical settings, site and extent of infection, and co-morbidities [12]. IL-6 is a biomarker in septic patients [93, 94], however, IL-6 levels are also upregulated in other non-infectious diseases [95, 96]. Of note, organ dysfunction often occurs during the process of sepsis such as sepsis-induced cardiac dysfunction and sepsis-associated pulmonary injury. Therefore, considering the limitations of the current biomarkers, it is necessary to develop novel biomarkers, especially those that can represent the specific organ dysfunction.

### Potential biomarkers for sepsis

Non-coding RNAs have been detected in different tissues or organs suggesting that they may be potential biomarkers for sepsis and sepsis-related organ dysfunction (Supplementary Tables 1 [79-83, 97-100] and 2 [18, 21, 22, 73, 85, 101-120]). Nowadays, the clinical sample which is suitable for detect non-coding RNAs is blood from patients, including serum, plasma, and blood leucocytes. Extracellular miRNAs exit in blood which could be quantified rapidly in a clinical setting, unlike microbial cultures that are time consuming [24]. Such property makes it possible to use miRNAs as biomarkers for the diagnosis of sepsis in clinic. As highlighted, different miRNAs are differentially expressed in sepsis. For example, studies showed that miR-16, miR122, and miR133a are increased in serum of septic patients [21, 106, 121], whereas the expression of some miRNAs, such as miR-25 and miR-181b is decreased [107, 116]. In addition, a recent study reported that the clinical accuracy of miRNA-25 for sepsis diagnosis was better than CRP and PCT (area under ROC curve =0.806, 0.676 and 0.726, for miRNA-25, CRP and PCT, respectively) [107]. The decrease in level of miRNA-25 was correlated with the severity of sepsis, SOFA score, CRP and PCT level [107]. Furthermore, a similar study showed that a cut-off point set at -1.89, miR-233 yielded a specificity of 100% and a sensitivity of 80%, while at a cut-off point set at -2.98, miR-146a yielded a specificity of 100% and a sensitivity of 63.3% [119]. These two miRNAs might serve as new biomarkers for sepsis with high specificity and sensitivity [119]. Additionally, miRNAs were reported to be related with prognosis in patients with sepsis. The study conducted by Vasilescu et al found that miR-150 levels were significantly reduced in plasma samples of sepsis patients and correlated with the level of disease severity measured by the SOFA scores [120]. MiR-150 was found to control c-Myb expression *in vivo* in a dose-dependent manner over a narrow range of miRNA and c-Myb concentrations, and this dramatically affected lymphocyte development and response [122], which could strengthen the functional significance of miR-150 downregulation in sepsis patients. Besides, the study conducted by Wang et al showed that a combination of sepsis stage, SOFA scores, and miR-574-5p could provide 78.13% sensitivity and 91.84% specificity of predictive capability for the death of septic patients [123]. Therefore, miRNAs could play a critical role in diagnosis and predictive capability for prognosis of sepsis.

### Potential biomarkers reflecting sepsis-induced cardiac dysfunction in sepsis

Sepsis-induced cardiac dysfunction is characterized by impaired myocardial contractility and reduced ejection fraction [124]. However, the pathophysiology of sepsis-induced myocardial dysfunction is unclear and there is currently no specific drug to reverse sepsis-induced myocardial dysfunction [125]. Several studies have showed that miRNAs have a potential role in sepsis-induced cardiac dysfunction. For example, miR-233 could be repressed and therefore generate an inflammatory response at multiple levels, subsequently inducing myocardial depression in a severe septic mouse model [109]. Additionally, Wang *et al.* showed that inhibition of miR-155 protected against LPS-induced cardiac dysfunction [101]. Furthermore, miR-146a can attenuate sepsis-induced cardiac dysfunction by preventing NF-κB activation and reduce inflammatory cell infiltration [57]. A recent study showed that LPS could inhibit miR-499 expression, which leads to cardiomyocyte apoptosis through the Bcl-2 family apoptotic pathway [103]. Except miRNAs, lncRNA also has been proved to be involved in the process of sepsis-induced cardiac dysfunction. In cardiomyocytes from septic mice, the lncRNA HOTAIR was found to be significantly upregulated and had a positive regulation on p65 phosphorylation and NF-kB activation [79]. Therefore, several non-coding RNAs have been identified that potentially mediate sepsis-induced cardiac dysfunction. These new non-coding RNAs may be novel diagnostic circulating biomarkers and have the potential to be developed into new intervention targets for patients prone to develop sepsis-induced cardiac dysfunction.

### Potential biomarkers representing sepsis-induced pulmonary injury

A previous review demonstrated that the treatment of underlying sepsis and identification of patients at risk of acute respiratory distress syndrome (ARDS) is of great importance in septic patients [126]. At present, there are no specific diagnostic biomarkers for sepsis-induced ARDS. MiRNAs may be involved in regulating sepsis-induced pulmonary injury, for example, Ying *et al.* showed that MiR-127 modulated macrophage polarization and promoted lung inflammation and injury by activating the JNK pathway [18], suggesting that miRNAs may be involved in sepsis-induced pulmonary injury. However, few studies have investigated the association between sepsis-induced pulmonary injury and non-coding. Further studies are needed to detect how non-coding RNAs influence pulmonary injury and to determine the regulatory mechanisms involved.

### Potential biomarkers regarding to sepsis-associated coagulopathy and endothelial dysfunction

Thrombocytopenia has become one of the most common abnormalities in patients with severe sepsis [127, 128]. A recent study showed that septic patients with thrombocytopenia had increased plasma and miRNAs expression levels of IL-18 and decreased expression of miR-130a, suggesting that miRNAs were involved in the pathophysiology of sepsis-associated thrombocytopenia and hence, a potential biomarker [102]. In addition, a clinical trial conducted by Wang *et al.* demonstrated that miR-122 levels were significantly higher in patients of the abnormal coagulation group than the normal coagulation group [121]. However, it is still unknown how the miRNAs affect coagulation and further studies are warranted to identify more miRNAs as biomarkers for coagulopathy.

Endothelial inflammation plays a critical role in the pathogenesis of sepsis [129]. A study discovered that LPS-induced endothelial dysfunction was mediated through the Slit2-Robo4 pathway and downregulation of Slit2 reduced the expression of miR-218 [110]. Moreover, miR-147b was involved in endothelial protection through degrading ADAM15 (a mediator regulating endothelial permeability) mRNA [111]. Taken together, this suggests that various miRNAs might be involved and could be novel biomarkers for sepsis-associated endothelial dysfunction.

### Potential biomarkers for sepsis-associated liver dysfunction or kidney dysfunction

A growing body of evidence has proved that miRNAs and/or lncRNAs participate in liver and kidney dysfunction during sepsis. For example, the expression of miR-142-3 increased in a rat cecal ligation and puncture model, thereby reducing adenylyl cyclase 9 expression in liver macrophages [130]. Similarly, the expression of miR-122 was upregulated in liver injury [108, 118]. Jia *et al.* also showed that knockdown of miR-21 upregulated its target effectors programmed cell death protein 4 and phosphatase and tensin homolog deleted on chromosome 10 expression, resulting in an increase in apoptosis and exacerbated LPS-induced acute kidney injury [105]. Notably, sequencing of RNA extracted from exposure of human proximal tubular epithelial cells to the plasma of critically ill septic patients demonstrated that a lncRNA, linc-ATP13A4-8, was significantly upregulated on exposure to plasma of septic patients [80]. Nonetheless, more research is required to explore organ-specific non-coding RNAs in liver or kidney and evaluate their credibility and validity as biomarkers for sepsis.

## PROMISE AND CHALLENGE IN USING NON-CODING RNAS AS BIOMARKERS AND THERAPEUTIC TARGETS

Increasing evidence suggests that non-coding RNAs play important roles in the regulation of pathophysiological processes of sepsis and sepsis-associated organ dysfunction, therefore they may be novel biomarkers and therapeutic targets [14]. However, their clinical application has not been evaluated and may encounter many challenges.

Firstly, non-coding RNAs as biomarkers is in its infancy. Although studies investigating the relationship between non-coding RNAs and sepsis have used RT-PCR and next-generation sequencing and microarray analyses [115, 131, 132], most studies regarding non-coding RNA in sepsis are only at the experimental stage. Notably, it is common to report sensitivity, specificity and associated area under the curve between miRNAs and sepsis [107, 119, 123, 133], but no studies have assessed the feasibility of selecting lncRNA/circRNA as novel biomarkers. Furthermore, the evaluation of the aforementioned association has only been performed in a relatively small number of studies, so reporting bias may exist due to differences in the area, population and race among studies. Large-scale clinical trials are warranted to identify more precise non-coding RNAs biomarkers for sepsis. Additionally, numerous studies have detected non-coding RNAs representing sepsis, but highly specific biomarkers for sepsis-associated organ dysfunction, such as sepsis-induced pulmonary injury, are relatively limited. The development of tissue-specific or organ-specific biomarkers would be helpful for the early treatment and intervention of organ failure, which may improve the survival rate of sepsis. Clearly, genome-wide profiling of miRNAs expression distinguished septic from non-septic patients. Nonetheless, prediction of the likelihood of a non-septic patient developing sepsis may be more clinically relevant to reduce mortality and morbidity in critical care units [14]. Additionally, as sepsis and septic shock are clinical emergency, further studies should detect whether non-coding RNAs could be used as biomarkers for early diagnosis and early prognosis of the diseases, and also compare economical aspect of non-coding RNAs with other methods.

Secondly, although many studies using cell and animal models have showed that miRNAs modulators be used in the fight against sepsis, considerable challenges must be overcome in order to successfully translate these approaches into clinical practice. Recent studies demonstrated that antisense miRNA inhibitors could be used to improve specific diseases in animal models and could offer novel therapeutic targets [134, 135]. Anti-miRNAs have been proved safe and already successful in clinical phase 2 studies [136]for the treatment of liver disease. The evaluation of the feasibility of miRNAs as septic therapy predominantly employs two approaches, use of antagomir or miRNA mimics [14]. However, sepsis and septic shock are different from some chronic diseases, because they are the medical emergencies. Therefore, the first challenge and problem to be considered is their delivery route. Besides, the expression of some miRNAs is organ-specific, so, further studies should focus on how to target specific organs without influencing others. According the latest guidelines [3], treatment often requires different types of medication at the same time. Therefore, the interaction between anti-miRNAs and such medications is also required for the management of septic patients. Moreover, with respect to clinical trials, there are striking inter-study variances of miRNA-regulation patterns in different cohorts of sepsis patients, which are most likely due to a lack in standardization in the sample collection, data normalization and analysis [25]. Therefore, a consensus on the optimal normalization strategy for miRNAs analysis from serum is required for more precise results.

Thirdly, the investigation of lncRNA/circRNA in sepsis may reveal the molecular mechanisms of sepsis. However, lncRNAs and circRNAs can exhibit diverse functions, for example, lncRNAs can regulate chromatin state through cis-regulation or trans-regulation, act as molecular scaffolds to organize protein complexes [36] and function as miRNA sponges to influence the action of miRNAs. Furthermore, some lncRNAs may act through more than one mechanism of action. Although limited molecular mechanistic studies have proved lncRNA/circRNA are involved in sepsis, it is unclear how lncRNA/circRNA influence the process of sepsis. Further studies could determine whether lncRNA/circRNA are organ-specific, potentially identifying new biomarkers and therapeutic targets.

As highlighted, if the above problems and challenges can be overcome, non-coding RNAs are attractive “next generation” biomarkers. With the in-depth understanding of the mechanism of sepsis, non-coding RNAs could also become the novel therapeutic targets in the future.

## CONCLUSIONS

In conclusion, increasing evidence has proved that non-coding RNAs are involved in the regulation of the pathophysiological process of sepsis. Although there are many problems and challenges regarding the application of non-coding RNAs clinical practice to be addressed, they have the potential to be new biomarkers and novel therapeutic targets for sepsis and septic shock. It is essential to further explore tissue- or organ-specific non-coding RNAs that might be more meaningful for targeted therapy of sepsis. In addition, future research should also focus on elucidating their molecular mechanisms, to further our understanding of the disease process. Non-coding RNAs may provide a new insight into sepsis and the development of mediators of non-coding RNAs may have a promising future.

## SUPPLEMENTARY MATERIALS TABLES







## Abbreviations

ARDS: acute respiratory distress syndrome
circRNAs: circular RNAs
CRP: C-reaction protein
DGCR: DiGeorge Syndrome Critical Region 8
IL-6: interleukin-6
lncRNAs: long non-coding RNAs
LPS: lipopolysaccharide
miRNAs: microRNAs
PCT: procalcitonin
RISC: RNA-induced silencing complex
TLR: toll-like receptor
TNF-α: tumor necrosis factor-α
