# Supplementary material for: Non-coding RNA: a potential biomarker and therapeutic target for sepsis
Source: Oncotarget. 2017 Oct 10;8(53):91765–78. doi: 10.18632/oncotarget.21766 (PMC5710963; doi:10.18632/oncotarget.21766)
Supplement: Supplementary file 1 [file oncotarget-08-91765-s001.pdf]

Non-coding RNA: a potential biomarker and therapeutic target for sepsis

SUPPLEMENTARY MATERIALS

Supplementary Table 1: Different expression and potential biomarkers of lncRNAs for sepsis.

See Supplementary File 1

Supplementary Table 2: Different expression and potential biomarkers of microRNAs for sepsis.

See Supplementary File 2
