# Supplementary material for: Non-coding RNA: a potential biomarker and therapeutic target for sepsis
Source: Oncotarget. 2017 Oct 10;8(53):91765–78. doi: 10.18632/oncotarget.21766 (PMC5710963; doi:10.18632/oncotarget.21766)
Supplement: Supplementary file 2 [file oncotarget-08-91765-s002.docx]

| **Author [Ref]** | **Year** | **Country** | **Study Sample** | **Specimen** | **Target** | **lncRNAs** | **Main Results** |
| --- | --- | --- | --- | --- | --- | --- | --- |
| Ma et al [97] | 2017 | US | RAW264.7 mouse macrophage cells  BV-2 mouse microglia  cells | macrophages | NF-kB pathway | lincRNA-Tnfaip3 | lincRNA-Tnfaip3 acts as a coactivator for the transcription of inflammatory genes in innate immune cells |
| Wang et al [98] | 2017 | China | mice | lung  T cells | N/A | lncRNAs | Airway allergic inflammation can cause alterations of lncRNAs expressed in mice and they are involved in the  alleviation of iPSC-MSC airway inflammation |

**Supplementary Table 1: Different expression and potential biomarkers of lncRNAs for sepsis**

**(Continued)**

**Supplementary Table S1. (Continued)**

| **Author [Ref]** | **Year** | **Country** | **Study Sample** | **Specimen** | **Target** | **lncRNAs** | **Main Results** |
| --- | --- | --- | --- | --- | --- | --- | --- |
| Yu et al [99] | 2017 | China | 6 permanent atrial fibrillation patients (pmAF)  6 healthy controls | lymphocytes | TNF signaling pathway  NF-kappaB signaling pathway  Toll-like receptor pathway  NOD-like receptor pathway | lncRNAs | Highly-expressed LncRNA1, LncRNA2 and LncRNA3 are closely associated with immunity and inﬂammation |
| Wu et al [79] | 2016 | China | mice | cell | NF-κB pathway | lncRNA-HOTAIR | ↑in cardiomyocytes from sepsis mice |
| Singh et al [82] | 2016 | Canada | Human umbilical vein endothelial cells(HUVECs) | cell | FoxO signaling pathways  TNF signaling pathways  Calcium signaling pathways  MAPK signaling  pathways  PPAR signaling pathways | lncRNAs | Of the 30,584 lncRNAs screened, 871 were significantly upregulated and 1068 significantly downregulated (p<0.05) in response to LPS |

**(Continued)**

**Supplementary Table S1. (Continued)**

| **Author [Ref]** | **Year** | **Country** | **Study Sample** | **Specimen** | **Target** | **lncRNAs** | **Main Results** |
| --- | --- | --- | --- | --- | --- | --- | --- |
| Sun et al [100] | 2016 | China | Pancreatic MIN6 β-cells | cell | NF-kappa B signaling pathway  Toll-like receptor signaling pathway  MAPK signaling pathway Jak-STAT signaling pathway  Hippo signaling pathway RIG-I-like receptor signaling pathway | lncRNAs | 444 upregulated and 279 downregulated lncRNAs were detected in MIN6 cells exposed to proinﬂammatory cytokines |
| Lin et al [80] | 2015 | US | Human proximal tubular epithelial cells (PTECs) | cell | HIF-1α pathway | MIR210HG  linc-ATP13A4-8  linc-KIAA1737-2 | ↑  ↑  ↑in cells treated with plasma from septic patients with acute kidney injury |

**(Continued)**

**Supplementary Table S1. (Continued)**

| **Author [Ref]** | **Year** | **Country** | **Study Sample** | **Specimen** | **Target** | **lncRNAs** | **Main Results** |
| --- | --- | --- | --- | --- | --- | --- | --- |
| Liu et al [81] | 2014 | US | N/A | blood | NF-κB pathway | lncRNAs | Of a stringent set of 4,284 lincRNAs, about 11–22% were expressed with 201 and 56 lincRNAs modulated by LPS in blood or adipose, respectively |
| Cui et al [83] | 2014 | UK | Human monocytic cell line THP-1  Human peripheral blood mononuclear cells (PBMCs)  Human umbilical vein endothelial cells (HUVECs) | cell | N/A | lnc-IL7R | ↑in LPS-treated cells and could regulate inflammatory regulation |

Note: ↑: upregulated ; ↓: downregulated ; ─: no difference; N/A: not available.
