# Supplementary material for: Non-coding RNA: a potential biomarker and therapeutic target for sepsis
Source: Oncotarget. 2017 Oct 10;8(53):91765–78. doi: 10.18632/oncotarget.21766 (PMC5710963; doi:10.18632/oncotarget.21766)
Supplement: Supplementary file 3 [file oncotarget-08-91765-s003.docx]

**Supplementary Table 2: Different expression and potential biomarkers of microRNAs for sepsis**

| **Author [Ref]** | **Year** | **Country** | **Study Sample** | **Specimen** | **Target** | **microRNAs** | **Main Results** |
| --- | --- | --- | --- | --- | --- | --- | --- |
| Ng et al [85] | 2016 | Malaysia | RAW264.7 (mouse embryonic fibroblast,MEF)  THP-1 cells | cell | ICAM-1 mRNAs | mcircRasGEF1B | mcircRasGEF1B might protect cells against microbial infection |
| Wang et al [101] | 2016 | China | 25 patients with septic cardiac dysfunction  21 sepsis patients without cardiac dysfunction | plasma | miR-155/Pea15a pathway | miR-155 | ↑in patients with  septic cardiac dysfunction |
| Cui et al [102] | 2016 | China | 28 severe sepsis patients with  thrombocytopenia  32 severe sepsis patients without thrombocytopenia | blood | IL-18 | miR-130a | ↓in severe sepsis patients with thrombocytopenia |
| Jia et al [103] | 2016 | China | Neonatal rat  ventricular myocytes | myocytes | miR-499SOX6/PDCD4-BCL-2 family pathway | miR-499 | ↓in response  to LPS stimulation |

**(Continued)**

**Supplementary Table S2. (Continued)**

| **Author [Ref]** | **Year** | **Country** | **Study Sample** | **Specimen** | **Target** | **microRNAs** | **Main Results** |
| --- | --- | --- | --- | --- | --- | --- | --- |
| Gao et al [104] | 2015 | US | mice | heart tissues | NF-κB pathway | miR-146a | miR-146a attenuates sepsis-induced  cardiac dysfunction |
| Jia et al [105] | 2015 | China | mice | serum  kidney slices | NF-κB pathway | miR-21 | ↑when Xenon protects against septic acute kidney injury |
| Wang et al [106] | 2015 | China | 46 neonates with sepsis  41 neonates with respiratory infection/  pneumonia(controls) | serum | TLR4 and IRAK1 | miR-15a  miR-15b  miR-16  miR-206  miR-223  miR-378  miR-451 | ↑  ─  ↑  ─  ─  ─  ─ in sepsis |

**(Continued)**

| **Author [Ref]** | **Year** | **Country** | **Study Sample** | **Specimen** | **Target** | **microRNAs** | **Main Results** |
| --- | --- | --- | --- | --- | --- | --- | --- |
| Ying et al [18] | 2015 | China | mice | blood | JNK pathway | miR-127 | miR-127 modulates macrophage polarization and promotes lung inflammation and injury |
| Yao et al [107] | 2015 | China | 70 patients with sepsis  30 patients with SIRS | blood | NADPH oxidase 4 (NOX4) | miR-21  miR-25  miR-203  miR-423-5p  miR-513a-5p  miR-503 | ─ in sepsis  ↓  ─  ─  ─  ─ |
| Roderburg et al [108] | 2015 | Germany | 138 ICU patients with sepsis  85 ICU patients without sepsis  76 healthy controls | serum | N/A | miR-122 | ↑when liver injury is present |
| Wang et al [109] | 2014 | US | mice | blood | IL-6,TNF-α | miR-223  miR-223 | ↓  ↓in severe septic mouse hearts |

**Supplementary Table S2. (Continued)**

**(Continued)**

**Supplementary Table S2. (Continued)**

| **Author [Ref]** | **Year** | **Country** | **Study Sample** | **Specimen** | **Target** | **microRNAs** | **Main Results** |
| --- | --- | --- | --- | --- | --- | --- | --- |
| Zhao et al [110] | 2014 | US | Human umbilical vein endothelial cells (HUVECs)  Human microvascular endothelial cells (HMVECs,adult dermis) | endothelial  cells | Slit2-Robo4 pathway | miR-218 | ↓when endothelial  dysfunction |
| Chatterjee  et al [111] | 2014 | US | Human lung microvascular endothelial cells (HLMECs)  Human umbilical vein endothelial cells (HUVECs) | endothelial cells | ADAM15 | miR-147b | miR-147b might protect barrier function in human vascular endothelial cells |
| Tacke et al [21] | 2014 | Germany | 138 ICU patients with sepsis  85 ICU patients without sepsis  76 healthy control | serum | N/A | miR-133a | ↑in sepsis |

**(Continued)**

**Supplementary Table S2. (Continued)**

| **Author [Ref]** | **Year** | **Country** | **Study Sample** | **Specimen** | **Target** | **microRNAs** | **Main Results** |
| --- | --- | --- | --- | --- | --- | --- | --- |
| Wang et al [112] | 2014 | China | 126 surviving sepsis patients  106 non-surviving sepsis patients  24 healthy controls | serum | N/A | miR-122  miR-193b  miR-483-5p  miR-574-5p | ↑  ↑  ↑  ↑in sepsis, surviving= non-surviving |
| Cheng et al [113] | 2013 | Canada | mice | serum | NF‐kB, AP‐1 ,MAPK/early growth response (EGR) pathways | miR-146 family | ↑induced by  proinflammatory  cytokines and acts to inhibit vascular inflammation |
| Wang et al [114] | 2013 | China | 14 sepsis patients  14 non-sepsis-SIRS patients | plasma | NF-κB/IRAK1/TRAF6/miR-146a loop | miR-146a | ↓in sepsis patients |
| Ma et al [115] | 2013 | UK | 23 sepsis patients  22 SIRS patients  21 healthy controls | blood | N/A | miR-150  miR-4772 family | ↓  ↑in sepsis patients |

**(Continued)**

**Supplementary Table S2. (Continued)**

| **Author [Ref]** | **Year** | **Country** | **Study Sample** | **Specimen** | **Target** | **microRNAs** | **Main Results** |
| --- | --- | --- | --- | --- | --- | --- | --- |
| Roderburg et al [22] | 2013 | Germany | 138 ICU patients with sepsis  85 ICU patients without sepsis  76 healthy controls | serum | c-Myb signaling  CXCR4 | miR-150 | (1) ↑MiR-150 is associated with an favorable outcome in patients with critical illness with or without sepsis  (2) ↓MiR-150 serum levels showed a strong and significant correlation with  decreased renal function |
| Li et al [73] | 2013 | US | N/A | blood | PMNs and macrophages | miR-466l | ↑(non-surviving＞  surviving＞  non-sepsis) |
| Sun et al [116] | 2012 | US | 36 sepsis patients  17 ICU patients without sepsis | plasma | NF-κB pathways | miR-181b | ↓in sepsis patients |

**(Continued)**

**Supplementary Table S2. (Continued)**

| **Author [Ref]** | **Year** | **Country** | **Study Sample** | **Specimen** | **Target** | **microRNAs** | **Main Results** |
| --- | --- | --- | --- | --- | --- | --- | --- |
| Wang et al [117] | 2012 | China | 117 surviving sepsis patients  97 non-surviving sepsis patients | serum | N/A | miR-15a  miR-16  miR-122  miR-193  miR-223  miR-483-5p | ↓  ↑  ↓  ↓  ↑  ↓in surviving sepsis patients |
| Waidmann  et al [118] | 2012 | Germany | 89 treatment-naive patients with chronic hepatitis B  19 healthy controls | serum | N/A | miR-122 | ↑strongly correlated with serum HBs antigen concentration |
| Wang et al [119] | 2010 | China | 50 sepsis patients  30 SIRS patients  20 healthy individuals | serum | NF-κB pathway | miR-146a  miR-223 | ↓in sepsis patients  ↓in sepsis patients |
| Vasilescu et al [120] | 2009 | Romania | 17 sepsis patients  32 healthy controls | blood | TNF-alpha, IL-10, and IL-18 | miR-150  miR-182  miR-342-5p  miR-486 | ↓  ↑in sepsis patients  ↓  ↑ |

Note: ↑: upregulated ; ↓: downregulated ; ─: no difference; N/A: not available.
